# Could the radiographic image quality be affected by the excessive use of the photostimulable phosphor plate?

**DOI:** 10.1590/0103-6440202305225

**Published:** 2023-03-06

**Authors:** Débora Costa Ruiz, Amanda Farias Gomes, Rocharles Cavalcante Fontenele, Francisco Haiter, Deborah Queiroz Freitas, Francisco Carlos Groppo

**Affiliations:** 1 Department of Oral Diagnosis, Piracicaba Dental School, University of Campinas, Piracicaba, SP, Brazil; 2 Department of Physiological Sciences, Piracicaba Dental School, University of Campinas, Piracicaba, SP, Brazil

**Keywords:** dental digital radiography, photostimulable phosphor plate, radiation exposure, X-rays

## Abstract

The study aimed to assess the influence of excessive use of a photostimulable phosphor plate (PSP) on the density, image noise, and contrast of radiographs. For that, radiographs of an acrylic block were acquired with a PSP of the Express intraoral system to assess the density and image noise. Initially, five images were obtained and exported (first group). After 400 exposures to X-rays and scannings of PSP, other five images were obtained and exported (second group). The same procedure was done after 800 (third group), 1200 (fourth group), 1600 (fifth group), and 2000 acquisitions (sixth group), resulting in 30 images to be evaluated. The mean and standard deviation of the gray values were calculated for the images using the ImageJ software. For contrast analysis, radiographs of an aluminum step-wedge were acquired with a new PSP following the same acquisition intervals. The percentage of contrast variation was calculated. Another two unused PSP receptors were employed to evaluate the method’s reproducibility. The comparison of the results among the acquisition groups was performed with one-way Analysis of Variance (α=0.05). Intraclass Correlation Coefficient (ICC) assessed the reproducibility of the receptors. Image noise did not differ among the groups (*p*>0.05). There was a slight increase in density after 400 acquisitions and a slight difference in contrast for all acquisition groups without a pattern of increase or decrease (*p*<0.05). ICC showed excellent reliability for the methods. Therefore, excessive PSP use slightly affected the radiograph’s density and contrast.

## Introduction

In dentistry, digital systems are divided in photostimulable phosphor plates (PSP) and solid-sensors ^1^. PSP receptors are distinguished from solid sensors by the thin structure, flexibility, and absence of an electrical cord ^1^ ^-^ ^4^. Other advantages of PSP receptors are the sizes available (similar to conventional films) and the wider dynamic range ^5^ ^,^ ^6^. Furthermore, manufacturers of PSP systems allege these devices can be used countless times as long as they are not physically damaged ^7^.

Previous studies reported that the image quality of the PSP was affected by its excessive use since a decrease in the mean of gray values (i.e. an increase in radiographic density) happened after several exposures to X-rays and scannings ^8^ ^,^ ^9^. The density of the resulting image was the only parameter evaluated and, although a change in density was reported in these previous studies, the number of acquisitions was not significant ^9^. In addition, the authors could not define exactly after how many exposures to X-rays and scannings these changes started to happen ^8^.

The longevity of a PSP is still not yet defined, what is known is that this receptor should be replaced in cases of bending or scratches ^7^. The authors of the present study hypothesized that the excessive use of the PSP receptor could not only affect the image density but also affect others parameters concerning the image quality, such as noise and contrast. Consequently, if this influence is detected, the radiographic diagnosis can be potentially impaired, making the receptor disposable after a certain number of acquisitions. 

According to the best of our knowledge, there are no studies evaluating the influence of excessive use of a PSP on these mentioned parameters. Therefore, the current study aimed to evaluate the influence of the excessive use of a PSP receptor on radiographic image quality by the assessment of the radiograph’s density, image noise, and contrast.

## Materials and Methods

Considering that the current study is based in an *in vitro* model, no ethical approval was required. To evaluate the influence of the excessive use of a PSP receptor, density, image noise, and contrast were measured using 4 unused sizes 2 PSP (2 for density and image noise, and other 2 for contrast).

Initially, to evaluate density and image noise, one unused size 2 PSP from the Express digital system (Instrumentarium Dental Inc., Milwaukee, WI, USA) and its respective native CliniView software (Instrumentarium Imaging, Tuusula, Finland) were used to obtain the radiographs. An acrylic block phantom (3.0 cm x 4.0 cm x 2.0 cm) was positioned in front of the PSP during all X-rays exposures for objective evaluation. The Focus X-ray unit (Instrumentarium, Tuusula, Finland) was adjusted to 70 kV, 7 mA, and an exposure time of 0.125 s. An acrylic holder with a fixed locator ring standardized the acquisitions (horizontal and vertical angulations of 0° and 90°, respectively; a focus-receptor distance of 40 cm and an acrylic block phantom-receptor distance of 0.5 cm). 

To promote the excessive use of the PSP receptor, a total of 2005 radiographic acquisitions were obtained (i.e. 2005 X-rays exposures plus scannings). First, five radiographs of the acrylic block phantom were acquired and exported, representing the first group. Then, 395 radiographs of the same phantom were obtained only to achieve an excessive use of this receptor, thus they were not used in the analyses. After a total of 400 acquisitions, five more radiographs were obtained and exported, thus representing the second group of radiographs tested. Subsequently, after 800 (third group), 1200 (fourth group), 1600 (fifth group), and 2000 acquisitions (sixth group), a group of five new radiographs were obtained and exported, resulting in 30 radiographs to be evaluated (six groups tested × five radiographs per group). The radiographs were acquired in a low-light room and the PSP receptor was immediately scanned after exposure (at a maximum of 10 seconds between the exposure to X-rays and the PSP scanning). During the acquisition of the radiographs, the PSP receptor was sheathed in a protective cardboard envelope provided by the manufacturer of the PSP.

The reproducibility of the receptors was evaluated using a second unused size 2 PSP of the same commercial brand aforementioned. For this purpose, five radiographs of the acrylic block were acquired and exported. Afterward, two groups of five radiographs were obtained and exported after 400 and 800 exposures and scannings of the PSP, totaling 15 radiographs and representing 50% of the groups previously evaluated (three groups tested × five radiographs per group). The radiographs obtained with the second PSP receptor followed the same parameters acquisitions applied to the first PSP and the images acquired (with the first and second PSP receptors) were obtained in 28 days since 100 radiographs were acquired daily. 

All 45 images (30 images obtained with the first PSP and 15 images obtained with the second PSP) were exported in 8-bit TIFF (Tagged Image File Format) and evaluated using the ImageJ software (National Institutes of Health, Bethesda, MD, USA) by one oral and maxillofacial radiologist with five years of experience in performing objective analysis in a silent and low-light environment. Two image quality parameters were measured for each radiograph: density and image noise. 

**Figure 1:**
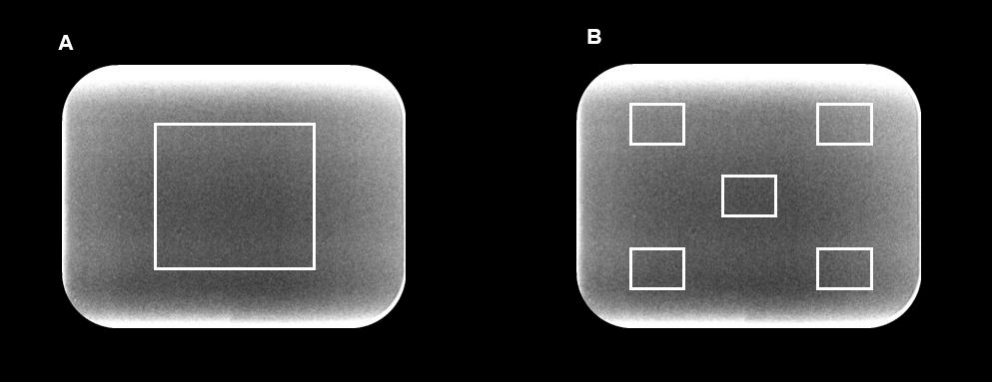
A. Density: Mean of gray values of the center ROI. B. Image noise: Average of the standard deviation of gray values of the five ROIs. ROIs, regions of interest

First, for the density analysis, a square region of interest (ROI) (Figure 1-A), located in the center of the image and covering 16% of its total area, was positioned to determine the mean of gray values ^10^. Second, to measure the image noise, one ROI (4 x 4 mm) was positioned in the center of the image. Based on its location, another four ROIs of the same size were symmetrically distributed in the upper and lower corners of the radiograph (Figure 1-B). Subsequently, to analyze the overall noise of the image, the average of the standard deviation (SD) of gray values obtained for all ROIs was calculated ^11^ ^,^ ^12^. Finally, the macro function available in the software was employed to standardize the position of the ROIs for all images evaluated. One hundred and twenty days after the image assessment ended, the same evaluator assessed the entire sample for a second time point to evaluate the intra-examiner reproducibility.

To evaluate contrast, radiographic images were acquired with another unused PSP receptor of the same commercial brand applied for evaluation of density and image noise. These acquisitions were performed using the same X-ray device and exposure protocols previously described. To do so, an aluminum step-wedge with the increasing thickness (2, 4, 6, 8, 10, and 12 mm) was placed horizontally in front of the PSP. The groups of acquisitions were the same applied to the aforementioned analyses. Therefore, this procedure resulted in 30 radiographs of the aluminum step-wedge being evaluated. A second unused PSP was also used to evaluate the reproducibility of the receptors, resulting in 15 more radiographs acquired and exported.

**Figure 2:**
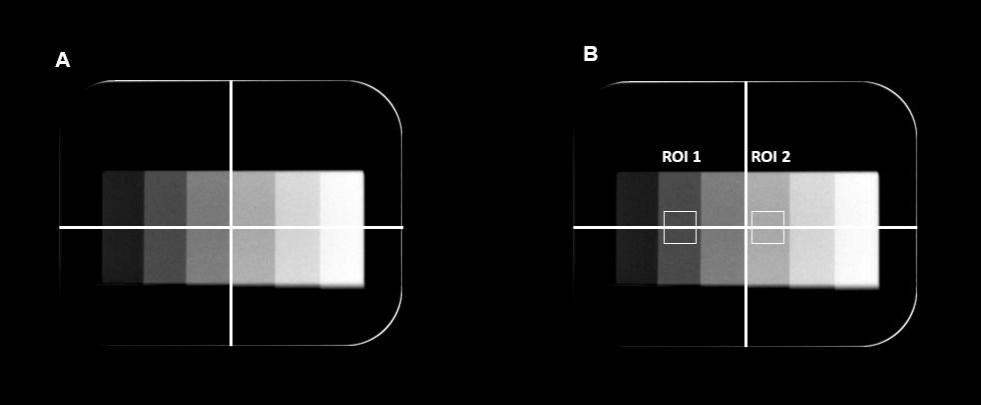
Image contrast analysis. A, in the center of the aluminum step-wedge, a line was drawn, dividing it equally. Then, a line perpendicular to the first one was determined. The 4 mm-thick (second) and the 8 mmthick (fourth) steps were selected for analysis. B, square ROIs were positioned in these steps and centralized on the perpendicular line. ROIs, regions of interest

All 45 radiographs were exported and analyzed using the ImageJ software. In this analysis, the percentage of contrast variation was calculated. First, a line was drawn in the center and parallel to the step-wedge. Then, another line (perpendicular to the first one) was drawn to equally divide the image of the aluminum step-wedge. Subsequently, one square ROI (16 × 16 mm) was positioned in the center of the second step (ROI 1), and another ROI of the same size was placed in the center of the fourth step (ROI 2) (Figure 2). The macro function was used to standardize the ROIs position. The percentage of contrast variation was set according to the formula ^13^:

$$\frac{\;(Mean\;of\;ROI2-Mean\;of\;ROI1)\;x\;100}{Mean\;of\;ROI2}$$

The same oral and maxillofacial radiologist performed this evaluation in a silent and low-light environment. Finally, the intra-examiner reproducibility was evaluated by the entire sample assessment, as previously explained for the density and image noise analyses.

One-way Analysis of Variance (ANOVA) compared the results of each image quality parameter (density, image noise, and contrast) with posthoc Tukey test. The significance level was set at 5% (*p*<0.05). In addition, Intraclass Correlation Coefficient (ICC) calculated the reproducibility of the PSP receptors. The same statistical test was used to evaluate the reliability of the image analysis within each measurement assessed. The null hypothesis considered that the excessive use of a PSP does not influence its objective image quality. All statistical analyses were performed using the SPSS 23.0 (SPSS Inc., Chicago, IL, USA) software.

## Results

According to Table 1, the ICC result showed a perfect or almost perfect reproducibility between the PSP receptors for all parameters evaluated. Regarding the reliability of the image assessment, an almost perfect agreement (ICC=0.99) was achieved for all analyses.

**Table 1:** ICC between PSP receptors

Image quality parameters	ICC
Density	1.00
Image noise	0.99
Contrast variation	0.99

%, percentage; ICC, Intraclass Correlation Coefficient

Table 2 shows the results concerning density, image noise, and contrast analyses among the different image acquisition groups. Although there were no differences for image noise values in any group of acquisitions (p>0.05), there was a statistically significant (*p*<0.05) slight increase in the radiographic density (i.e. lower mean of gray values of the larger and center ROI) after a total of 400 exposures to X-rays and scannings of the PSP, representing darker images. Regarding contrast, a significant change was noticed for all acquisition groups without a pattern of increase or decrease among the six groups evaluated (*p*<0.05), with higher values observed in the fifth group. 

**Table 2:** Mean of gray values from the center ROI, average of SD of gray values and contrast variation (%) from the ROIs among the different groups of image acquisitions

Group of acquisitions	Density (Mean of the larger and center ROI)	Image noise (Average of the SD of gray values of 5 ROIs)	Contrast variation (%)
First group	106.95 (0.76) A	9.40 (0.17)	65.30 (0.59) CD
Second group	105.32 (0.65) B	9.43 (0.12)	64.91 (0.20) DE
Third group	105.23 (0.40) B	9.37 (0.23)	66.09 (0.20) B
Fourth group	105.69 (1.08) AB	9.26 (0.26)	65.78 (0.34) BC
Fifth group	104.97 (0.82) B	9.34 (0.21)	68.10 (0.10) A
Sixth group	105.75 (0.90) AB	9.19 (0.23)	64.62 (0.17) E
*p values*	*0.01*	*0.51*	*0.01*

SD, standard deviation ; %, percentage;

Different letters indicate significant difference among the groups of acquisitions (p<0.05) according to the one-way Analysis of Variance.

## Discussion

The knowledge of the impact of several uses of a PSP receptor is necessary because when image quality is compromised, the radiographic diagnosis can be negatively affected ^1^ ^,^ ^14^ ^,^ ^15^. Previous studies have reported that multiple exposures to X-rays and scanning caused a decrease in the pixel gray values of the resulting images ^8^ ^,^ ^9^. According to the present findings, a slight increase in radiograph density, no significant changes in the image noise, and a slight change in contrast among the six acquisitions groups were observed. This result is the opposite of the authors’ hypothesis, who believed that the excessive use of a PSP would negatively influence other parameters not yet evaluated, such as image noise and contrast. 

A decrease in the mean of gray values calculated on a center ROI covering a wide area of the PSP represents an increase in the radiographic density ^11^ ^,^ ^12^. Regarding the contrast variation, these values varied among the acquisitions groups without a pattern of increase or decrease. Although these statistical differences suggest that, the excessive use of a PSP receptor influences the density and contrast of the resulting images, their clinical implication should be carefully interpreted. Minimal statistical differences were indicated as significant because there was a consistency in the values obtained in the acquisition groups, and, consequently, low SD values. Therefore, by analyzing the data in the tables, it can be seen that the values are very close, and those differences would probably not be noticed by a human’s eye ^16^.

Previous studies that motivated the present research also employed objective analyses to evaluate the effects of excessive PSP use on image quality ^4^ ^,^ ^8^ ^,^ ^9^. Ergün et al. analyzed the image quality of radiographs acquired with the Digora Optime system after 200 acquisitions through a subtraction method and the measurement of the mean of gray values ^9^. A significant decrease in the mean of gray values was observed when the twentieth image was subtracted from the baseline image, agreeing with the present results. However, in this previous research, only one radiograph per group was evaluated. In contrast, in the present study, five radiographs per group were analyzed, which is more representative of an experimental group. These other four repetitions eliminate the bias of the results due to a potential oscillation in the tube current during PSP receptor exposure to X-rays or the possibility of the findings being only a random result. 

Furthermore, Souza-Pinto et al. exposed and scanned the same PSP system used in the current study 1800 times and evaluated the mean of gray values in standardized ROIs ^8^. A significant increase in density was observed when the values obtained in the ROIs from the first and last acquisitions were compared, also agreeing with the present findings. However, this aforementioned study could not define the number of acquisitions needed to cause these significant changes. Differently, the methodology used in the current study could identify that the density increase happens after 400 exposures to X-rays and scannings of the PSP receptor (i.e. before 1800 acquisitions).

In contrast, Matsuda et al. acquired 1000 radiographs of an aluminum step-wedge with a Digora Optime PSP system ^4^. Their results revealed no significant difference among the mean of gray values located in predetermined ROIs on the image of the steps of the aluminum device, partly disagreeing with the present findings. The possible reason for this disagreement is the number of acquisitions and the type of density analysis employed. While the current study evaluated the mean of gray values using a homogeneous acrylic block phantom in 2000 acquisitions, Matsuda et al. evaluated the density in radiographs of an aluminum step-wedge after 1000 exposures to X-rays and scannings. Although there are differences in the interpretation of data and statistical analysis, our understanding of low clinical significance is similar and agrees with this aforementioned study. 

An increase in the pixel values was previously reported in PSP receptors that had a delay in their scanning process ^17^. This aspect could compromise the present study results. Therefore, the PSP was scanned at a maximum of 10 seconds between the exposure to X-rays and the PSP scanning during all radiographic acquisitions. Besides, to standardize the methodology applied, a homogeneous acrylic block phantom was positioned in front of the PSP in all acquisitions obtained to evaluate density and image noise. Therefore, obtaining a homogeneous interaction with radiation was possible, resulting in a homogeneous image, which is crucial for the image quality analyses performed ^11^.

A second PSP (same size and commercial brand) was used in each objective analysis (density, image noise, and contrast variation) to evaluate the methods’ reliability by measuring ICC. The results revealed excellent reliability in all parameters tested, meaning that different PSP receptors of the same commercial brand showed a similar pattern regarding image quality parameters assessed. However, these results cannot be generalized to other commercial brands of PSP receptors. Thus, future studies should evaluate different brands to testify if these findings are applicable to other digital radiographic systems. In addition, the authors of this research encourage future investigations evaluating the influence of excessive use of a PSP on the accuracy of diagnostic tasks (such as caries lesions, root fractures, or root resorptions).

The excessive use of a PSP receptor did not influence the image noise and slightly influenced the density and contrast of the images acquired. Therefore, according to the findings, intraoral PSP receptors can be used up to a least 2000 times without impairing the quality of the resulting images.
